# Network Coding Approaches for Distributed Computation over Lossy Wireless Networks

**DOI:** 10.3390/e25030428

**Published:** 2023-02-27

**Authors:** Bin Fan, Bin Tang, Zhihao Qu, Baoliu Ye

**Affiliations:** 1 Key Laboratory of Water Big Data Technology of Ministry of Water Resources, Hohai University, Nanjing 211100, China; 2 School of Computer and Information, Hohai University, Nanjing 211100, China

**Keywords:** distributed computing, coded computation, network coding, lossy wireless network, BATS codes

## Abstract

In wireless distributed computing systems, worker nodes connect to a master node wirelessly and perform large-scale computational tasks that are parallelized across them. However, the common phenomenon of straggling (i.e., worker nodes often experience unpredictable slowdown during computation and communication) and packet losses due to severe channel fading can significantly increase the latency of computational tasks. In this paper, we consider a heterogeneous, wireless, distributed computing system performing large-scale matrix multiplications which form the core of many machine learning applications. To address the aforementioned challenges, we first propose a random linear network coding (RLNC) approach that leverages the linearity of matrix multiplication, which has many salient properties, including ratelessness, maximum straggler tolerance and near-ideal load balancing. We then theoretically demonstrate that its latency converges to the optimum in probability when the matrix size grows to infinity. To combat the high encoding and decoding overheads of the RLNC approach, we further propose a practical variation based on batched sparse (BATS) code. The effectiveness of our proposed approaches is demonstrated by numerical simulations.

## 1. Introduction

In recent years, due to the proliferation of computationally intensive applications at the wireless edge, such as federated learning [1] and image recognition [2], wireless distributed computing has drawn great interest [3,4], where large-scale computational tasks are carried out by a cluster of wireless devices collaboratively. Meanwhile, due to the inherent randomness of wireless environment, wireless distributed computing systems are facing multiple challenges. One main challenge is called the straggler issue, where computing devices often experience unpredictable slowdown or even dropout during computation and communication, which can lead the computational task to much larger latency or even failure [5]. Another challenge is the packet-loss issue, where the packets can be lost during transmission due to severe channel fading of wireless networks.

In this paper, we consider a typical wireless distributed computing system consisting of multiple worker nodes and a master node. We focus on distributed matrix multiplication y=Ax, which forms the core of many computation-intensive machine learning applications, such as linear regression, and aims at tackling the two above challenges. One common approach to mitigate the effect of stragglers is providing redundancy through replication [6,7,8], which has been widely used in large distributed systems such as MapReduce [9] and Spark [10]. However, this kind of *r*-replication strategy can only tolerate *r* stragglers, and using a larger *r* increases the computation redundancy, which can lead to poor performance.

Recently, Lee et al. [11] firstly introduced coding-based computation framework, and then proposed an (n,k) maximum-distance-separable (MDS) code approach, such that the master node can recover the desired result from the local computation results of any *k* out of *n* worker nodes. Based on this, Das et al. further proposed a fine-grained model such that the partial results of stragglers can be leveraged. However, MDS codes fail to make full use of the partial work done by stragglers. Ferdinand et al. [12] and Kiani et al. [13] proposed approaches to make use of stragglers by allocating more fine-grained computing tasks to each worker. Very recently, Mallick et al. [14] proposed the use of rateless codes such as LT codes [15] and Raptor codes [16] and demonstrated that a rateless coding approach can achieve an asymptotically optimal latency. However, all these approaches assumed that the communication between each worker node and the master node is reliable and can only lead to inferior performance in wireless distributed computing.

In fact, the packet-loss issue has been widely investigated in communication networks, and the existing approaches roughly belong to two categories. The first is automatic repeat-request (ARQ) based, which employs feedback-based retransmissions to combat packet loss. It has been adopted by Han et al. [17] in a MDS-code-based wireless distributed computing system. However, the feedbacks from the master node can increase the computation latency significantly due to the inherent delays of feedback, especially when the communication traffic between worker nodes and the master node is large. The other is forward error correction (FEC)-based, employing error-correcting code to combat packet losses. Traditional FEC approaches mainly focus on achieving reliable transmission over each communication link, but in the context of distributed matrix multiplication, the objective is to recover the desired computation result. How to tackle both the straggler issue and the packet-loss issue for distributed matrix multiplication in wireless distributed computing system remains an open problem.

In this paper, by leveraging the linearity of matrix multiplication, we show how network coding [18] can be applied to solve the two issues efficiently in a joint manner. The main contributions of this paper are summarized as follows:

- We first propose a random linear network coding (RLNC) [19] based approach. In this approach, the matrix A to be multiplied is first split into multiple submatrices $A_{1},\ldots ,A_{k}$, and each worker node is assigned multiple submatrices, each of which is a random linear combination of the $A_{1},\ldots ,A_{k}$. Each worker node multiplies each assigned submatrix with the input x, and it generates random linear combinations of submatrix-vector products that have been created for transmission. Once receiving enough packets with independent global encoding vectors, the master node can recover the desired result Ax by Gaussian elimination. We model the computation and communication process as a continuous-time trellis, and by conducting a probabilistic analysis of the connectivity of the trellis, we theoretically show that the latency of RLNC approach converges to the optimum in probability when the matrix size grows to infinity.
- Since RLNC approach has high encoding and decoding costs, we further propose a practical variation of RLNC approach based on batched sparse (BATS) code [20] and show how to optimize the performance of the BATS approach.
- We conducted numerical simulations to evaluate the proposed RLNC and BATS approaches. The simulation results show that both approaches can overcome the straggler issue and the packet-loss issue effectively and achieve near-optimal performance.

The reminder of the paper is organized as follows. Section 2 introduces the system model. Section 3 and Section 4 introduce the RLNC approach and the BATS approach, respectively. Section 5 presents the numerical evaluation results. Finally, Section 6 concludes.

## 2. System Model

### 2.1. Coding-Based Wireless Distributed Computation

**Figure 1:**
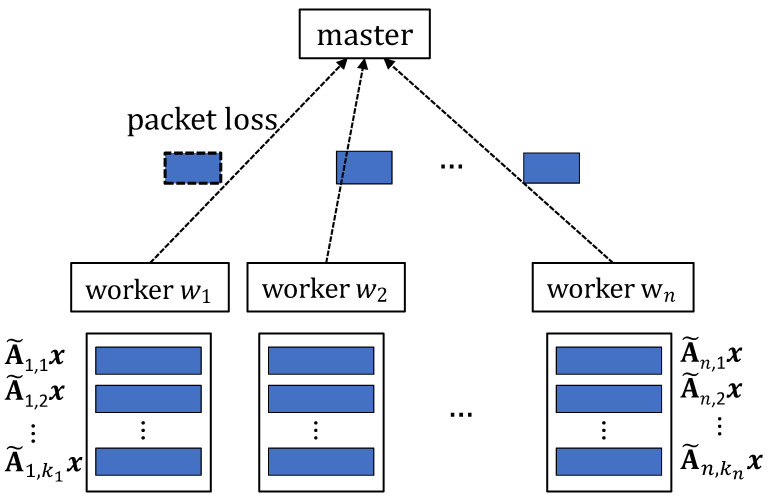
Illustration of the wireless distributed computing system for matrix multiplication.

As shown in Figure 1, we consider a heterogeneous, wireless distributed computing system consisting of a master node and *n* heterogeneous worker nodes. These worker nodes, denoted by $w_{1},w_{2},\ldots ,w_{n}$, are connected wirelessly to the master node. We focus on the matrix-vector multiplication problem, whose goal is to compute the result y=Ax for a given matrix $A\in R^{m\times d}$ and an arbitrary vector $x\in R^{d\times 1}$, where R is a set of real numbers. Our results can be directly extended to matrix-matrix multiplication, where x is a small matrix.

In order to mitigate the effect of unpredictable node slowdown during computation and communication, we consider an error-correcting code based computing framework which consists of four components:

- **Encoding before computation**: The matrix A is first split along its rows equally into *k* submatrices $A_{1},\ldots ,A_{k}$, i.e., $A^{T}=\left[A_{1}^{T}\;A_{2}^{T}\;\cdots \;A_{k}^{T}\right]$. Without loss of generality, here we assume that m/k is an integer. These submatrices are encoded into more submatrices using an error-correcting code, which are further placed on worker nodes. The submatrices assigned to worker node $w_{i}$ are denoted as ${\tilde{A}}_{i,1},{\tilde{A}}_{i,2},\ldots ,{\tilde{A}}_{i,k_{i}}$, where $k_{i}$ is the number of submatrices assigned to $w_{i}$. Here, we emphasize that, in many applications, such as linear regression, this encoding will be used for multiple computations with different inputs x [11], so that the encoding is often required to be executed before the arrival of any x.
- **Computation at each worker node**: When an input x is arrived at the master node, the master node will broadcast x to all these worker nodes. Once worker node $w_{i}$ receives x, it will compute ${\tilde{A}}_{i,1}x,{\tilde{A}}_{i,2}x,\ldots ,{\tilde{A}}_{i,k_{i}}x$ in a sequential manner.
- **Communication from each worker node**: During the computation, each worker node also keeps on sending its local computation results to the master node in some manner. For this, each submatrix-vector product which is a vector of length m/k is encapsulated into a packet. We assume that the communication link between worker *i* and the master node can be modeled as a packet erasure channel, where each packet is erased independently with probability $\varepsilon_{i}$. In order to combat these packet losses, each worker node can transmit its local computation results using a coding based approach.
- **Decoding at the master node**: Once the master node receives enough information, it will recover the desired result y=Ax and notify all the worker nodes to stop the computation.

### 2.2. Delay Model

In this paper, we mainly focus on minimizing the latency, which is the time required by the wireless computing system so that the result y=Ax can be successfully decoded at the master node by aggregating the results sent from the worker nodes. For the characterization of the latency, we consider the following two models, one for computation delay and the other for communication delay.

As in [14], we consider a computation delay model as follows. The computation delay at each worker node $w_{i}$ consists of two parts. The first is an initial setup time before $w_{i}$ starts to perform any submatrix-vector multiplication, denoted by $X_{i}$, which is assumed to follow an exponential distribution with rate $\lambda_{i}$. The second is a constant time for calculating each submatrix-vector product, which is denoted by $\tau_{i}$. Hence, the delay for computing *r* submatrix-vector products by $w_{i}$ is $X_{i}\;+\;\tau_{i}r$.

In order to characterize the straggling effect during the communication, we model the communication time of a packet from worker node *i* to the master node as a shifted-exponential distribution with rate $\mu_{i}$ and shift parameter $\theta_{i}$. Additionally, the communication times of all packets are mutually independent. The model has also been adopted by [17,21].

## 3. A Network Coding Approach

In order to combat the straggling effects during both computation and communication and the packet losses during communication, in this section, we propose a random linear network coding (RLNC)-based approach and show that it can achieve optimal latency performance in the asymptotic sense, i.e., when the number of rows of A goes to infinity, when the overheads incurred are ignored. A practical version of this approach is given in the next section.

### 3.1. Description

We describe the RLNC based approach based on the computing framework given in Section 2.1:

**Encoding before computation:** In the RLNC-based approach, each submatrix ${\tilde{A}}_{i,j}$ assigned to worker node $w_{i}$ is a random linear combination of $A_{1},\ldots ,A_{k}$; i.e.,

(1) $${\tilde{A}}_{i,j}=\sum_{e=1}^{k}c_{i,j,e}A_{e},\;j=1,2,\ldots ,k_{i}$$

where $c_{i,j,e}$ is chosen randomly and independently according to a standard normal distribution. Since this encoding approach is rateless, $k_{i}$ can be arbitrarily large.

**Computation at each worker node:** When the worker node $w_{i}$ receives an input x, it starts to compute the local results ${\tilde{y}}_{i,1}={\tilde{A}}_{i,1}x,{\tilde{y}}_{i,2}={\tilde{A}}_{i,2}x,\ldots ,{\tilde{y}}_{i,k_{i}}={\tilde{A}}_{i,k_{i}}x,$ in a sequential manner.

**Communication from each worker node:** For each packet transmission starting at time *t*, the worker node $w_{i}$ will generate a linear combination of all the local computation results in hand as

(2) $${\hat{y}}_{i,t}=\sum_{j=1}^{d_{i}\left(t\right)}c_{j}^{\prime }{\tilde{y}}_{i,j},$$

where $d_{i}\left(t\right)$ is the number of local results that have been computed before time *t* by $w_{i}$. Here, $\left(c_{1}^{\prime },\ldots ,c_{d_{i}\left(t\right)}^{\prime }\right)$ is referred to as the local encoding vector of ${\hat{y}}_{i,t}$.

**Decoding at the master node:** Due to the linearity of matrix-vector multiplication, we can see that

(3) $$\begin{array}{cc} {\hat{y}}_{i,t} & =\sum_{j=1}^{d_{i}\left(t\right)}c_{j}^{\prime }{\tilde{y}}_{i,j}=\sum_{j=1}^{d_{i}\left(t\right)}c_{j}^{\prime }{\tilde{A}}_{i,j}x\\ \; & =\sum_{j=1}^{d_{i}\left(t\right)}c_{j}^{\prime }\sum_{e=1}^{k}c_{i,j,e}A_{e}x\\ \; & =\sum_{e=1}^{k}\left(\sum_{j=1}^{d_{i}\left(t\right)}c_{j}^{\prime }c_{i,j,e}\right)A_{e}x \end{array}$$

i.e., each packet received by the master node is a linear combination of $A_{1}x,A_{2}x,\ldots ,A_{k}x$. Here,

(4) $$\left(\sum_{j=1}^{d_{i}\left(t\right)}c_{j}^{\prime }c_{i,j,1},\sum_{j=1}^{d_{i}\left(t\right)}c_{j}^{\prime }c_{i,j,2},\ldots ,\sum_{j=1}^{d_{i}\left(t\right)}c_{j}^{\prime }c_{i,j,k}\right)$$

is referred to as the global encoding vector of ${\hat{y}}_{i,t}$. Hence, when the master node receives enough packets that have *k* linearly independent global encoding vectors, it can recover the desired results $A_{1}x,A_{2}x,\ldots ,A_{k}x$ by Gaussian elimination.

**Overhead:** Our RLNC approach suffers from its high encoding and decoding complexities, just like RLNC for communication. More specifically, in our approach, the encoding cost per submatrix is O(k·m/k·d)=O(md), and the total decoding cost is $O\left(k^{3}\;+\;k^{2}\cdot m/k\right)=O\left(k^{3}\;+\;mk\right)$. We can see that the encoding cost is high, but the encoding can been done before any computation and just once, which can be used for computing Ax as many times as possible with different x. Meanwhile, the decoding cost is also high when *k* is large, but it is independent of *d*, the number of columns of A. Thus, when *d* is very large, the decoding cost at the master node can be much lower than the computation cost at each worker node. In addition, the decoding at the master node can be done in an incremental fashion using Gauss–Jordan elimination, which can further reduce the decoding latency.

Note that the global encoding vector is required by the master node for decoding. To achieve this efficiently, we use a pseudo-random number generator to generate the local encoding vector for each transmitted packet and append the random seed. The number of local results are computed for the packet. Then, the master node can get the global encoding vectors according to (3). In this way, the coefficient overhead is negligible, which is opposite to the traditional RLNC for communication networks.

**Remark** **1.** *Lin et al. [22] have also applied RLNC in distributed training on mobile devices. They used RLNC to create coded data partitions among mobile devices so as to tolerate computational uncertainties, and their main purpose is to reduce the need to exchange data partitions across mobile devices. Differently from [22], the use of RLNC in this paper is for straggler mitigation and packet-loss tolerance in a joint manner, while leveraging the computation and communication capabilities of all worker nodes.*

**Remark** **2.** *Since random linear network coding is performed over the field of real numbers as opposed to a finite field, the entries of generated matrices could be very large numbers, leading the whole computation to be numerically unstable. In fact, this issue is present in any coded distributed computation over the field of real numbers and is not just limited to our approaches. There are two basic approaches to dealing with this issue. One is to use very small coefficients to avoid the emergence of large numbers, which is possible, as the encoding operations are also linear with these coefficients in our proposed approach. This is significantly different from the Reed–Solomon-code/polynomial-code-based approaches which have been widely adopted in coded distributed computation (see, e.g., [11,23]), as the coefficients are powers of evaluation points. In particular, the numerical instability issue for the RLNC approach is much less severe than that for Reed–Solomon-code/polynomial-code-based approaches, since Vandermonde matrices have exponentially large condition numbers. The other is to employ the finite field embedding technique [24,25], where the entries are quantized into number of finite digits and then embedded into a finite field. Nevertheless, both approaches incur numerical errors. How to guarantee numerical stability in coded distributed computation is still an open problem and requires further study.*

### 3.2. Latency Analysis

Let $r_{i}=\frac{1}{\theta_{i}\;+\;1/\mu_{i}}$, and $r_{i}^{\prime }=\min\left\{1/\tau_{i},r_{i}\left(1-\varepsilon_{i}\right)\right\}$. Define

(5) $$T_{0}=\frac{k}{\sum_{i=1}^{n}r_{i}^{\prime }}\;+\;\frac{\sum_{i=1}^{n}r_{i}^{\prime }X_{i}}{\sum_{i=1}^{n}r_{i}^{\prime }}.$$

The following result characterizes a upper bound of the latency of the proposed RLNC-based approach.

**Theorem** **1.** *For any constant δ>0, the latency of the proposed RLNC-based approach, denoted by $T_{\mathrm{RLNC}}$, satisfies*

(6) $$\lim_{k\to \infty }\mathrm{Pr}\left(T_{\mathrm{RLNC}}\leq \left(1\;+\;\delta \right)T_{0}\right)=1.$$

The following result establishes a lower bound on the latency of any scheme under the coding framework.

**Theorem** **2.** *For any scheme under the coding framework, the probability that its latency $T_{\mathrm{any}}$ is less than $T_{0}$ decays exponentially with k; i.e., for any constant δ>0, there exists some constant η>1 that does not depend on k, such that*

(7) $$\mathrm{Pr}\left(T_{\mathrm{any}}\geq \left(1-\delta \right)T_{0}\right)=1-O\left(\eta^{-k}\right).$$

From Theorems 1 and 2, it is straightforward to see that the proposed RLNC-based approach is asymptotically optimal. In the following, we will formally prove Theorems 1 and 2 by a connectivity analysis of a continuous-time trellis, which models the computation and communication processes.

**Figure 2:**
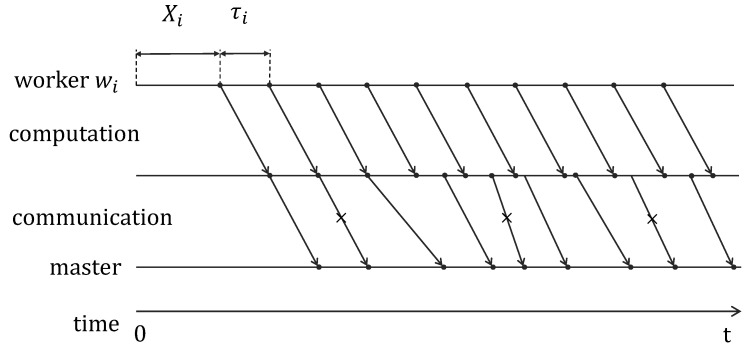
Illustration of a continuous-time trellis, $G_{i}^{\left(t\right)}$.

For any scheme under the coding framework, as illustrated in Figure 2, we model the computation and communication processes of each worker node $w_{i}$ up to time *t* using a continuous-time trellis ($G_{i}^{\left(t\right)}$) [26], where edges are classified into three types: computation edges, transmission edges and memory edges. Each computation edge models the computation of a submatrix-vector product. Suppose $w_{i}$ computes a submatrix-vector product from time $t_{0}$ to $t_{0}\;+\;\tau_{i}\leq t$. Then, two nodes, $w_{i}\left(t_{0}\right)$ and $w_{i}^{\prime }\left(t_{0}\;+\;\tau_{i}\right)$, will be introduced, and there is a computation edge from $w_{i}\left(t_{0}\right)$ to $w_{i}^{\prime }\left(t_{0}\;+\;\tau_{i}\right)$. Similarly, suppose a packet is transmitted from $w_{i}$ at time $t_{0}$ and received successfully by the master node at time $t_{1}\leq t$. Then, two nodes $w_{i}^{\prime }\left(t_{0}\right)$ and $m(t_{1})$, if they do not exist, will be introduced, and there is a transmission edge from $w_{i}^{\prime }\left(t_{0}\right)$ to $m_{i}\left(t_{1}\right)$. We also introduce nodes $w_{i}\left(0\right)$ and a node $m_{i}\left(t\right)$. Nodes $\left\{w_{i}\left(\cdot \right)\right\}$ are connected through the timeline, so are nodes $\left\{w_{i}^{\prime }\left(\cdot \right)\right\}$ and nodes $\left\{m_{i}\left(\cdot \right)\right\}$. The edges for such connections are called memory edges. Each computation edge and each transmission edge is associated with unit capacity, and each memory edge is associated with an infinity capacity. Finally, we construct a global continuous-time trellis $G^{\left(t\right)}$, which includes the union of all $G_{i}^{\left(t\right)}$ and two auxiliary nodes w(0) and m(t). In addition, there is an edge from w(0) to each $w_{i}\left(0\right)$ with an infinity capacity, and there is an edge from each $m_{i}\left(t\right)$ to m(t) with an infinity capacity.

The usefulness of the continuous-time trellis model is summarized in the following result.

**Proposition** **1.** *For any scheme that achieves latency of T, then the maximum flow from w(0) to m(T) in its continuous-time trellis $G^{\left(T\right)}$ must be least k. Moreover, for our RLNC approach, if the maximum flow from w(0) to m(T) in its continuous-time trellis $G^{\left(T\right)}$ is at least k, then the master node can recover the desired computation result at time T with probability one.*

**Proof.**  It is straightforward to see that the first part holds. The second part is inherited from the optimality of RLNC in communication networks [19] and the fact that all the operations are over the real field R. □

Now, we proceed to prove Theorems 1 and 2. We start by presenting some concentration results regarding the communication between worker nodes and the master node.

**Lemma** **1.** *Suppose $Y_{1},Y_{2},\ldots$ follow a shifted exponential distribution with rate μ and shift parameter θ independently. Then, for any constant δ>0, there exists some constant $\eta_{1}>1$, such that*

(8) $$\mathrm{Pr}\left(\left|\sum_{i=1}^{s}Y_{i}-\left(\theta \;+\;\mu^{-1}\right)s\right|>\delta \left(\theta \;+\;\mu^{-1}\right)s\right)=O\left(\eta_{1}^{-s}\right).$$

**Proof.**  The result can be proved by a Chernoff-like argument based on moment generating function [27].

The moment generating function of $Y_{i}$ is

(9) $$E\left[e^{-hY_{i}}\right]=\frac{\mu }{\mu \;+\;h}e^{-h\theta }$$

Hence,

(10) $$\begin{array}{cc} \; & \mathrm{Pr}\left(\sum_{i=1}^{s}Y_{i}<\left(1-\delta \right)\left(\theta \;+\;\mu^{-1}\right)s\right)\\ \; & =\mathrm{Pr}\left(e^{-h\sum_{i=1}^{s}Y_{i}}>e^{-h\left(1-\delta \right)\left(\theta \;+\;\mu^{-1}\right)s}\right)\\ \; & \leq \frac{E\left[e^{-h\sum_{i=1}^{s}Y_{i}}\right]}{e^{-h\left(1-\delta \right)\left(\theta \;+\;\mu^{-1}\right)s}}\\ \; & =\frac{\prod_{i=1}^{s}E\left[e^{-hY_{i}}\right]}{e^{-h\left(1-\delta \right)\left(\theta \;+\;\mu^{-1}\right)s}}\\ \; & =\frac{{\left(\frac{\mu }{\mu \;+\;h}e^{-h\theta }\right)}^{s}}{e^{-h\left(1-\delta \right)\left(\theta \;+\;\mu^{-1}\right)s}} \end{array}$$

where the inequality holds by applying the Markov’s inequality. Let $h=\frac{1}{\left(1-\delta \right)\left(\theta \;+\;\mu^{-1}\right)-\theta }-\mu$. We then have

(11) $$\begin{array}{cc} \; & \mathrm{Pr}\left(\sum_{i=1}^{s}Y_{i}<\left(1-\delta \right)\left(\theta \;+\;\mu^{-1}\right)s\right)\\ \; & \leq {\left(\frac{e^{\mu \left(1-\delta \right)\left(\theta \;+\;\mu^{-1}\right)-\theta }-1}{\mu \left(1-\delta \right)\left(\theta \;+\;\mu^{-1}\right)-\theta }\right)}^{-s}\\ \; & \leq {\left(\frac{e^{1-\delta (1\;+\;\theta \mu )}-1}{1-\delta (1\;+\;\theta \mu )}\right)}^{-s} \end{array}$$

By setting $\eta_{1}=\frac{e^{1-\delta (1\;+\;\theta \mu )}-1}{1-\delta (1\;+\;\theta \mu )}$, we get the desired result. □

For a scheme, let $N_{i}\left(t\right)$ ($N_{i}^{\prime }\left(t\right)$, resp.) be the number of packet transmissions (successful packet transmissions, resp.) from worker node $w_{i}$ to the master node during the time interval $\left(X_{i},X_{i}\;+\;t\right)$.

**Lemma** **2.** *For any scheme and any constant δ>0, there exists some constant $\eta_{2}>1$, such that*

(12) $$\mathrm{Pr}\left(N_{i}\left(t\right)\geq \left(1\;+\;\delta \right)r_{i}t\right)=O\left(\eta_{2}^{-t}\right).$$

**Proof.**  Let $Y_{1},Y_{2},\ldots ,Y_{N_{i}\left(t\right)}$ be i.i.d. shifted exponential random variables with rate $\mu_{i}$ and shift parameter $\theta_{i}$, and $s=\left\lceil \left(1\;+\;\delta \right)r_{i}t\right\rceil$. According to Lemma 1, there exist some constant $s=\left\lceil \left(1\;+\;\delta \right)r_{i}t\right\rceil \eta_{1}>1$ and $\eta_{2}=\eta_{1}^{\left(1\;+\;\delta \right)r_{i}}$ such that

(13) $$\begin{array}{cc} \; & \mathrm{Pr}\left(N_{i}\left(t\right)\geq \left(1\;+\;\delta \right)r_{i}t\right)\\ \; & \leq \mathrm{Pr}\left(\sum_{j=1}^{s}Y_{j}\leq t\right)\\ \; & \leq \mathrm{Pr}\left(\sum_{j=1}^{s}Y_{j}\leq \left(1-\frac{\delta }{1\;+\;\delta }\right)\left(\theta_{i}\;+\;\mu_{i}^{-1}\right)s\right)\\ \; & \leq \eta_{1}^{-s}=O\left(\eta_{2}^{-t}\right) \end{array}$$

□

**Lemma** **3.** *For any scheme and any constant δ>0, there exists some constant $\eta_{3}>1$ such that*

(14) $$\mathrm{Pr}\left(N_{i}^{\prime }\left(t\right)\geq \left(1\;+\;\delta \right)r_{i}\left(1-\varepsilon_{i}\right)t\right)=O\left(\eta_{3}^{-t}\right).$$

**Proof.**  Let *A* denote the event that $N_{i}\left(t\right)\geq \left(1\;+\;\delta /2\right)r_{i}t$. By the total law of probability,

(15) $$\begin{array}{cc} \; & \mathrm{Pr}\left(N_{i}^{\prime }\left(t\right)\geq \left(1\;+\;\delta \right)r_{i}\left(1-\varepsilon_{i}\right)t\right)\\ \; & =\mathrm{Pr}\left(N_{i}^{\prime }\left(t\right)\geq \left(1\;+\;\delta \right)r_{i}\left(1-\varepsilon_{i}\right)t|A\right)\mathrm{Pr}\left(A\right)\\ \; & \;\;\;+\;\mathrm{Pr}\left(N_{i}^{\prime }\left(t\right)\geq \left(1\;+\;\delta \right)r_{i}\left(1-\varepsilon_{i}\right)t|\bar{A}\right)\mathrm{Pr}\left(\bar{A}\right)\\ \; & \leq \mathrm{Pr}\left(A\right)\;+\;\mathrm{Pr}\left(N_{i}^{\prime }\left(t\right)\geq \left(1\;+\;\delta \right)r_{i}\left(1-\varepsilon_{i}\right)t|\bar{A}\right) \end{array}$$

According to Lemma 2, there exists some constant $\eta_{2}^{\prime }>1$ such that $\mathrm{Pr}\left(A\right)=O\left(\eta_{2}^{-t}\right)$. Let *N* be a binomial random variable with parameters $\left(1\;+\;\delta /2\right)r_{i}t$ and $1-\varepsilon_{i}$. Then, there exists some constant $\eta_{3}^{\prime }>1$ such that

(16) $$\begin{array}{cc} \; & \mathrm{Pr}\left(N_{i}^{\prime }\left(t\right)\geq \left(1\;+\;\delta \right)r_{i}\left(1-\varepsilon_{i}\right)t|\bar{A}\right)\\ \; & \leq \mathrm{Pr}\left(N\geq \left(1\;+\;\delta \right)r_{i}\left(1-\varepsilon_{i}\right)t\right)\\ \; & =O\left(\eta_{3}^{\prime -t}\right) \end{array}$$

where the second step follows by applying the Chernoff bound for a binomial random variable [27]. Finally, by letting $\min\left\{\eta_{3}^{\prime }=\eta_{2}^{\prime },\eta_{3}^{\prime }\right\}$, we have

(17) $$\mathrm{Pr}\left(N_{i}^{\prime }\left(t\right)\geq \left(1\;+\;\delta \right)r_{i}\left(1-\varepsilon_{i}\right)t\right)=O\left(\eta_{3}^{-t}\right)$$

□

**Lemma** **4.** *For any scheme, let $F_{i}\left(t\right)$ be the maximum flow from $w_{i}\left(0\right)$ to m(t) in its continuous-time trellis $G^{\left(t\right)}$. Then, for any constant δ>0, there exists some constant $\eta_{4}>1$ such that*

(18) $$\mathrm{Pr}\left(F_{i}\left(\left(1-\delta \right)T_{0}\right)\geq \frac{r_{i}^{\prime }k}{\sum_{j=1}^{n}r_{j}^{\prime }}\right)\leq O\left(\eta_{4}^{-k}\right).$$

**Proof.**  Let *B* be the event that $\left(1-\delta \right)T_{0}-X_{i}>\left(1-\delta /2\right)\frac{k}{\sum_{j=1}^{n}r_{j}^{\prime }}$. Then

(19) $$\begin{array}{cc} \mathrm{Pr}(B) & \leq \mathrm{Pr}\left(\left(1-\delta \right)T_{0}>\left(1-\delta /2\right)\frac{k}{\sum_{i=1}^{n}r_{i}^{\prime }}\right)\\ \; & =\mathrm{Pr}\left(\sum_{i=1}^{n}r_{i}^{\prime }X_{i}>\frac{\delta }{2(1-\delta )}k\right)\\ \; & \leq \mathrm{Pr}\left(\exists i\;s.\;t.\;r_{i}^{\prime }X_{i}>\frac{\delta }{2n(1-\delta )}k\right)\\ \; & \leq \sum_{i=1}^{n}\mathrm{Pr}\left(r_{i}^{\prime }X_{i}>\frac{\delta }{2n(1-\delta )}k\right)\\ \; & =\sum_{i=1}^{n}e^{-\frac{\lambda_{i}\delta }{2n\left(1-\delta \right)r_{i}^{\prime }}k}=O\left(\eta_{4}^{\prime -k}\right) \end{array}$$

for some constant $\eta_{4}^{\prime }>1$. By the total law of probability,

(20) $$\begin{array}{cc} \; & \mathrm{Pr}\left(F_{i}\left(\left(1-\delta \right)T_{0}\right)\geq \frac{r_{i}^{\prime }k}{\sum_{j=1}^{n}r_{j}^{\prime }}\right)\\ \; & =\mathrm{Pr}\left(F_{i}\left(\left(1-\delta \right)T_{0}\right)\geq \frac{r_{i}^{\prime }k}{\sum_{j=1}^{n}r_{j}^{\prime }}|A\right)\mathrm{Pr}\left(A\right)\\ \; & \;\;+\;\mathrm{Pr}\left(F_{i}\left(\left(1-\delta \right)T_{0}\right)\geq \frac{r_{i}^{\prime }k}{\sum_{j=1}^{n}r_{j}^{\prime }}|\bar{A}\right)\mathrm{Pr}\left(\bar{A}\right)\\ \; & \leq \mathrm{Pr}\left(A\right)\;+\;\mathrm{Pr}\left(F_{i}\left(\left(1-\delta \right)T_{0}\right)\geq \frac{r_{i}^{\prime }k}{\sum_{j=1}^{n}r_{j}^{\prime }}|\bar{A}\right) \end{array}$$

We consider two cases. In the first case, $\frac{1}{\tau_{i}}\leq r_{i}\left(1-\varepsilon_{i}\right)$. Thus, $r_{i}^{\prime }=\frac{1}{\tau_{i}}$. Since $F_{i}\left(t\right)$ cannot exceed the number of computation edges $\frac{t-X_{i}}{\tau_{i}}$, it is straightforward to check that

(21) $$\mathrm{Pr}\left(F_{i}\left(\left(1-\delta \right)T_{0}\right)\geq \frac{r_{i}^{\prime }k}{\sum_{j=1}^{n}r_{j}^{\prime }}|\bar{A}\right)=0.$$

Thus, $\mathrm{Pr}\left(F_{i}\left(\left(1-\delta \right)T_{0}\right)\geq \frac{r_{i}^{\prime }k}{\sum_{j=1}^{n}r_{j}^{\prime }}\right)=O\left(\eta_{4}^{\prime -k}\right)$. In the second case, $\frac{1}{\tau_{i}}>r_{i}\left(1-\varepsilon_{i}\right)$. Thus, $r_{i}^{\prime }=r_{i}\left(1-\varepsilon_{i}\right)$. Since $F_{i}\left(\left(1-\delta \right)T_{0}\right)\leq N_{i}^{\prime }\left(\left(1-\delta \right)T_{0}-X_{i}\right)$,

(22) $$\begin{array}{cc} \; & \mathrm{Pr}\left(F_{i}\left(\left(1-\delta \right)T_{0}\right)\geq \frac{r_{i}^{\prime }k}{\sum_{j=1}^{n}r_{j}^{\prime }}|\bar{A}\right)\\ \; & \leq \mathrm{Pr}\left(N_{i}^{\prime }\left(\left(1-\delta \right)T_{0}-X_{i}\right)\geq \frac{r_{i}^{\prime }k}{\sum_{j=1}^{n}r_{j}^{\prime }}|\bar{A}\right)\\ \; & \leq \mathrm{Pr}\left(N_{i}^{\prime }\left(\left(1-\delta /2\right)\frac{k}{\sum_{j=1}^{n}r_{j}^{\prime }}\right)\geq \frac{r_{i}^{\prime }k}{\sum_{j=1}^{n}r_{j}^{\prime }}\right)\\ \; & =O(\eta_{5}^{-k}) \end{array}$$

for some constant $\eta_{5}>1$, where the last step follows from Lemma 3. Thus, we can show that $\mathrm{Pr}\left(F_{i}\left(\left(1-\delta \right)T_{0}\right)\geq \frac{r_{i}^{\prime }k}{\sum_{j=1}^{n}r_{j}^{\prime }}\right)=O\left(\eta_{4}^{-k}\right)$ for constant $\eta_{4}=\min\left\{\eta_{4}^{\prime },\eta_{5}\right\}$. □

Now we are ready to prove Theorem 2.

**Proof**  (Proof of Theorem 2). For any scheme, since the maximum flow from w(0) to $m(\left(1-\delta \right)T_{0})$ in its continuous-time trellis $G^{\left(\left(1-\delta \right)T_{0}\right)}$ is equal to $\sum_{i=1}^{n}F_{i}\left(\left(1-\delta \right)T_{0}\right)$, according to Proposition 1, its latency $T_{\mathrm{any}}$ satisfies

(23) $$\begin{array}{cc} \; & \mathrm{Pr}(T_{\mathrm{any}}\leq \left(1-\delta \right)T_{0})\\ \; & \leq \mathrm{Pr}\left(\sum_{i=1}^{n}F_{i}\left(\left(1-\delta \right)T_{0}\right)\geq k\right)\\ \; & \leq \mathrm{Pr}\left(\exists i\;s.t.\;F_{i}\left(\left(1-\delta \right)T_{0}\right)\geq \frac{r_{i}^{\prime }k}{\sum_{j=1}^{n}r_{j}^{\prime }}\right)\\ \; & \leq \sum_{i=1}^{n}\mathrm{Pr}\left(F_{i}\left(\left(1-\delta \right)T_{0}\right)\geq \frac{r_{i}^{\prime }k}{\sum_{j=1}^{n}r_{j}^{\prime }}\right)\\ \; & =O(\eta_{4}^{-k}) \end{array}$$

where the last step follows from Lemma 4. □

Next, we turn to prove Theorem 1. For the RLNC approach and $t\geq X_{i}$, let $N_{i}\left(t,t\;+\;\Delta t\right)$ be the number of successful packet transmissions from worker node $w_{i}$ to the master node during the time interval (t,t+Δt). We have the following result.

**Lemma** **5.** *For any $t\geq X_{i}$,*

(24) $$\frac{N_{i}\left(t,t\;+\;\Delta t\right)}{\Delta t}\overset{P}{\to }r_{i}\left(1-\varepsilon_{i}\right),\;\mathrm{as}\;\Delta t\to \infty ;$$

*i.e., $N_{i}\left(t,t\;+\;\Delta t\right)/\Delta t$ converges to $r_{i}\left(1-\varepsilon_{i}\right)$ in probability when Δt goes to infinity, or equivalently, for any constant ϵ>0.*

**Proof.**  The result can be shown similarly to that of Lemma 3. □

**Lemma** **6.** *Let $F_{i}\left(t\right)$ be the maximum flow from $w_{i}\left(0\right)$ to m(t) in the continuous-time trellis $G^{\left(t\right)}$ of the RLNC approach. Then,*

(25) $$\frac{F_{i}\left(t\right)}{t-X_{i}}\overset{P}{\to }\min\left\{\frac{1}{\tau_{i}},r_{i}\left(1-\varepsilon_{i}\right)\right\}=r_{i}^{\prime },\;\mathrm{as}\;t\to \infty ,$$

**Proof.**  According to Theorem 1 of [26], Lemma 5 implies this result immediately. □

Now we can prove Theorem 1.

**Proof.**  (Proof of Theorem 1). According to Lemma 6, $F_{i}\left(T_{0}\right)\overset{P}{\to }\left(T_{0}-X_{i}\right)r_{i}^{\prime }$, as k→∞. Hence, $\sum_{i=1}^{n}F_{i}\left(T_{0}\right)\overset{P}{\to }k$ as k→∞. Since,

(26) $$\begin{array}{cc} \; & \frac{F_{i}\left(\left(1\;+\;\delta \right)T_{0}\right)}{F_{i}\left(T_{0}\right)}\\ \; & \geq \frac{F_{i}\left(\left(1\;+\;\delta \right)T_{0}\right)}{\left(1\;+\;\delta \right)T_{0}-X_{i}}\cdot \frac{\left(1\;+\;\delta \right)\left(T_{0}-X_{i}\right)}{F_{i}\left(T_{0}\right)}\overset{P}{\to }1\;+\;\delta , \end{array}$$

it is straightforward to check that

(27) $$\lim_{k\to \infty }\mathrm{Pr}\left(\sum_{i=1}^{n}F_{i}\left(\left(1\;+\;\delta \right)T_{0}\right)<k\right)=0.$$

According to Proposition 1, this implies that

(28) $$\lim_{k\to \infty }\mathrm{Pr}\left\{T_{\mathrm{RLNC}}\geq \left(1\;+\;\delta \right)T_{0}\right\}=0.$$

The proof is accomplished. □

## 4. BATS-Code-Based Approach

As mentioned earlier, despite its optimality, RLNC based approach suffers from its high encoding and decoding overheads. In this section, we propose a new approach based on batched sparse (BATS) code [20], which is a variation of RLNC having low encoding and decoding overheads.

### 4.1. Description

In the BATS-code-based approach, the *k* submatrices $A_{1},\ldots ,A_{k}$ are first encoded into $A_{1},\ldots ,A_{k},A_{k\;+\;1},\ldots ,A_{k^{\prime }}$ using a fixed-rate systematic erasure code (called a precode), where $k^{\prime }=\left(1\;+\;\epsilon \right)k$ and ϵ is a small positive constant (e.g., 0.02). BATS codes are rateless, as an infinite number of batches can be generated. The generation of each batch is as follows:

- Sample a degree deg according to a given degree distribution $\Psi =(\Psi_{1},\ldots ,\Psi_{D})$, where *D* is the maximum degree;
- Select deg distinct submatrices uniformly at random from $A_{1},\ldots ,A_{k},A_{k\;+\;1},\ldots ,A_{k^{\prime }}$;
- Generate *M* random linear combinations of the deg submatrices, which are referred to as a batch.

Based on BATS code, batches of submatrices are assigned to worker nodes, and each worker node performs the local computation on the basis of a batch, which consists of *M* submatrix-vector multiplications. In order to forward the computational result of a batch to the master node, each worker node will generate a number of packets, each of which is a random linear combination of the *M* submatrix-vector products corresponding to the batch. For decoding, the master node first recovers $A_{1}x,\ldots ,A_{k}x,A_{k\;+\;1}x,\ldots ,A_{k^{\prime }}x$ using Gaussian-elimination-based belief propagation (BP) decoding, and once any *k* or slightly more than *k* of $A_{1}x,\ldots ,A_{k}x,A_{k\;+\;1}x,\ldots ,A_{k^{\prime }}x$ are recovered, the master node can recover all these $A_{1}x,\ldots ,A_{k}x$ by decoding the precode. See [20] for more details.

**Overhead:** In the BATS-code-based approach, the encoding cost per submatrix is $O\left(deg\cdot \frac{m}{k}\cdot d\right)=O\left(\frac{md}{k}\right)$, and the total decoding cost is $O\left(\left(M^{3}\;+\;M^{2}\frac{m}{k}\right)\cdot \frac{k}{M}\right)=O(M^{2}k\;+$Mm). Clearly, both the encoding cost and decoding cost are much lower than for the RLNC approach, especially when *M* is a small constant (e.g., 8 or 16). As for the RLNC approach, the decoding cost is independent of *d*, and the coefficient overhead is negligible when leveraging the pseudo-random-number-generator-based approach.

**Remark** **3.** *There have been many other sparse variants of random linear network coding, including chunked codes (e.g., [28,29]), tunable sparse network coding (e.g., [30,31], and sliding-window coding (e.g., [32,33,34,35,36]). While many of these codes can also be applied, BATS codes are more suitable for this distributed computing scenario. On the one hand, BATS codes are rateless. Thus, all the worker nodes can keep on computing and forwarding local results to the master node before the whole computation is completed, as long as enough batches are placed on each worker node. In contrast, chunked codes (e.g., [28,29]) usually have fixed coding rates or require a lot of feedback from the master node. On the other hand, as mentioned in Section 2, in many applications, the step of encoding before computation is required to be performed before the arrival of any input x. In other words, this encoding step should be irrelevant to the uncertain computation and communication processes of worker nodes. However, differently from BATS codes, sliding-window codes are often generated on-the-fly and are not as suitable as BATS codes.*

### 4.2. Performance Optimization

The performance of BATS code heavily depends on how the *M* computation results of each batch are transmitted to the master node, and which degree distribution is used.

Suppose that worker node $w_{i}$ sends $Z_{i}$ coded packets to the master nodes for the computation results of each batch $B_{j}$. Let $H_{j}$ be a $Z_{i}\times M$ matrix, where each row corresponds to a transmitted packet. If the packet is successfully received by the master node, then the row is the local encoding vector. Otherwise, the row is zero-vector. Let $h_{i}=\left(h_{i,0},\ldots ,h_{i,M}\right)$ denote the rank distribution of $H_{j}$, where $h_{i,r}$ is the probability that $H_{j}$ has rank *r*. We can show that

(29) hi,r=∑ℓ=rubPr(Zi=ℓ)ℓr(1−εi)rεiℓ−r,r≤M−1∑ℓ=MubPr(Zi=ℓ)∑s=Mℓℓs(1−εi)sεiℓ−s,r=M.

where ub is an upper bound of $Z_{i}$. In order to maximize the transmission efficiency for BATS code, we apply the linear programming method [37] to optimize the distribution of $Z_{i}$:

(30) $$\begin{array}{cc} \max & \sum_{r=1}^{M}rh_{i,r}\\ \;s.t.\; & \sum_{\ell =0}^{ub}\mathrm{Pr}\left(Z_{i}=\ell \right)\ell \left(\theta_{i}\;+\;\mu_{i}^{-1}\right)\leq M\tau_{i}\\ \; & \sum_{\ell =0}^{ub}\mathrm{Pr}\left(Z_{i}=\ell \right)=1\\ \; & 0\leq \mathrm{Pr}\left(Z_{i}=\ell \right)\leq 1,\;\ell =0,\ldots ,ub \end{array}$$

Here, the objective is to maximize the expected rank. The first constraint stands for the expected time for transmitting $Z_{j}$ packets to the master node being no larger than the time for computing *M* submatrix-vector multiplications, and the last two constraints stand for $\mathrm{Pr}(Z_{i}=\ell ),\ell =0,\ldots ,ub$ being a probability distribution.

When the time goes to infinity, we can see that the proportion of batches whose computation results have been sent to the master node by worker node $w_{i}$ is $\frac{1/\tau_{i}}{\sum_{j=1}^{n}1/\tau_{j}}$. Hence, we can derive the empirical rank distribution h over all the batches done by worker nodes as

(31) $$\begin{array}{c} h=\sum_{i=1}^{n}\frac{1/\tau_{i}}{\sum_{j=1}^{n}1/\tau_{j}}h_{i}. \end{array}$$

Based on the empirical rank distribution, we can find a good degree distribution Ψ such that the BATS code can achieve a coding rate close to $\bar{h}/M$, where $\bar{h}$ is the expected value corresponding to the empirical rank distribution (c.f. [20]).

## 5. Performance Evaluation

In this section, we first evaluate the decoding cost incurred by our proposed approaches, and then we present simulations conducted to evaluate the overall computational performances of these approaches in comparison to some state-of-the-art approaches.

We first ran some experiments on a computer with an Intel(R) Core(TM) i7-10700 CPU 2.90 GHz and Python 3.7. In these experiments, the matrix A was 50,000 × *d*, where *d* ranged from 1000 to 16,000. Matrix A was split into 1000 sub-matrices of the same size, and each submatrix consisted of 50 rows so that each transmitted packet consisted of 50 real numbers. In the BATS-code-based approach, the batch size was set to eight. We simulated the decoding process and evaluated the decoding delays (in terms of second) of both the RLNC based approach and the BATS-code-based approach. The delay for the original matrix multiplication was also evaluated. The results are presented in Table 1.

Note that the decoding latencies of both the RLNC based approach and the BATS-code-based approach are irrelevant to *d*, and the latency for the original matrix multiplication grows linearly with *d*. From this table, we can see that even when d=1000, the decoding latency of the BATS-code-based approach is only about 1.58% of the latency of original computation, and when *d* grows larger, this latency becomes negligible. In contrast, when d=1000 or d=2000, the decoding cost of the RLNC based approach is prohibitive.

We also conducted simulations to evaluate the performances of our proposed approaches. In our simulations, the number of worker nodes was 10, and the settings of matrix A remained the same as above, except that the number of columns *d* was irrelevant in our simulations. We simulated four scenarios. In the first three scenarios, worker nodes were homogeneous, and the size relationship between computation time per submatrix-vector product and average communication time of a packet varied among these scenarios. In the last scenario, worker nodes were heterogeneous. The involved parameters of these scenarios are given as follows.

- Scenario I, where $\left(\lambda_{i},\tau_{i}\right)=\left(0.1,0.2\right)$, $\left(\mu_{i},\theta_{i}\right)=\left(20,0.05\right)$ and $\varepsilon_{i}=0.2$;
- Scenario II, where $\left(\lambda_{i},\tau_{i}\right)=\left(0.1,0.15\right)$, $\left(\mu_{i},\theta_{i}\right)=\left(10,0.05\right)$ and $\varepsilon_{i}=0.2$;
- Scenario III, where $\left(\lambda_{i},\tau_{i}\right)=\left(0.1,0.1\right)$, $\left(\mu_{i},\theta_{i}\right)=\left(10,0.1\right)$ and $\varepsilon_{i}=0.2$;
- Scenario IV, where for each worker i, parameters $\lambda_{i}$, $\tau_{i}$, $\mu_{i}$, $\theta_{i}$ and $\varepsilon_{i}$ were uniformly distributed at random over intervals [0.07, 0.2], [0.1, 0.3], [10, 20], [0.05, 0.2] and [0.1, 0.4], respectively.

For these scenarios, we evaluated the following five methods.

- **Uniform uncoded**, where the divided sub-matrices were equally assigned to 10 worker nodes—i.e., each worker node computed 100 sub-matrices.
- **Two-Replication**, where the divided sub-matrices were equally assigned to five worker nodes, and the computing tasks of these worker nodes were replicated at another five worker nodes.
- (10,8)**MDS code**, where the divided 1000 sub-matrices were encoded into 1250 sub-matrices and then equally assigned to 10 worker nodes.
- **LT code** [14], where the 1000 original sub-matrices were encoded using LT codes, and an infinite number of coded sub-matrices was assigned to each worker node.
- **RLNC**: The details are introduced in Section 3. The time cost of recoding and decoding operations was ignored.
- **BATS code**: The details are introduced in Section 4, and a batch size of eight was used.

While our proposed schemes tackle the packet-loss issue, the first four of the above schemes do not consider this issue at all. For these schemes, we used an ideal retransmission (IR) scheme for the first four schemes, where the worker nodes know whether a transmitted packet is lost or not immediately. This leads these schemes to perform better. In the following, we refer to the first four schemes as **Uncoded + IR**, **Rep + IR**, **(10,8)MDS + IR** and **LT + IR**, respectively.

**Figure 3:**
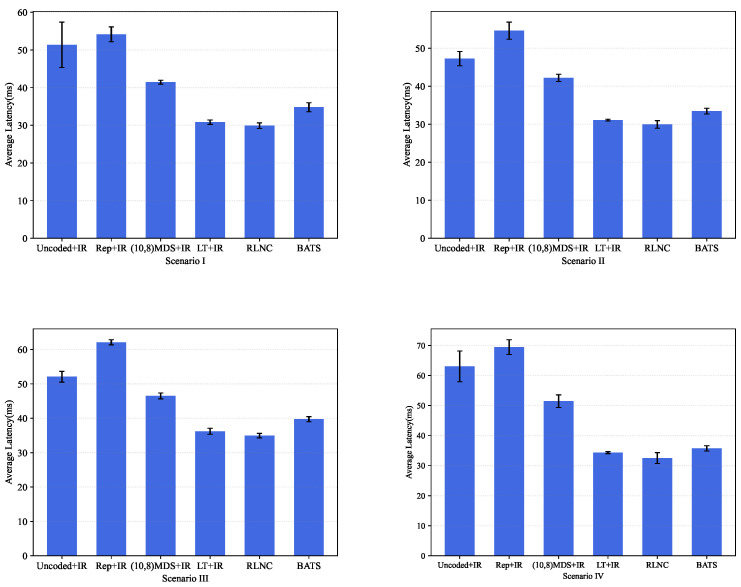
The latency performances of different approaches under four scenarios, where the error bar indicates the standard deviation.

The latency performance levels of these approaches under the four scenarios are plotted in Figure 3, where the decoding latency at the master node is ignored. From this figure, we observe the following.

- Among the first four schemes, LT + IR achieved the best performance for all four scenarios. Note that IR eliminates the packet-loss issue, and this result has also been demonstrated in [14], where only the straggler issue was considered. This is because LT codes can achieve near-perfect load balance among the worker nodes in the presence of stragglers.
- For all these scenarios, the proposed RLNC approach achieved the best latency performance among all these schemes. In particular, the performance of the RLNC approach was slightly better than that of LT + IR. Just like LT + IR, our RLNC approach also achieved near-perfect load balance among the worker nodes. Meanwhile, LT + IR incurred a small precode overhead, whereas the RLNC approach did not. This result also demonstrates the near-optimality of the RLNC approach.
- Our BATS approach performed much better than Uncoded + IR, Rep + IR, and (10,8) MDS + IR in all these scenarios, but slightly worse than LT + IR and RLNC. Since LT + IR assumes an ideal retransmission scheme, which is impractical, and the RLNC approach incurs high encoding and decoding costs, the BATS approach is much more practical.

In summary, both our RLNC approach and our BATS approach can overcome both the straggler issue and the packet-loss issue effectively and can achieve near-optimal performance in different scenarios when the number of columns *d* is large enough.

## 6. Conclusions

In this paper, we focused on addressing the straggler issue and the packet-loss issue jointly for distributed matrix multiplication in wireless distributed computing systems. We proposed an RLNC approach and proved its asymptotical optimality using a continuous-time-trellis-based argument. We further proposed a more practical variation of the RLNC approach based on BATS code. The effectiveness of both approaches was demonstrated through numerical simulations.

## Figures and Tables

**Table 1:** The decoding delays (in terms of second) of our proposed approaches in comparison with the delay of original matrix multiplication.

d=	1000	2000	4000	8000	16,000	32,000
matrix multiplication delay	34.16	69.59	138.69	280.86	550.43	1116.84
decoding delay (RLNC)	34.51	34.51	34.51	34.51	34.51	34.51
decoding delay (BATS)	0.54	0.54	0.54	0.54	0.54	0.54
